# The Role of Pathological Examination of the Liver in Medicolegal Autopsy: A Tertiary Care Center Study From North India

**DOI:** 10.7759/cureus.48131

**Published:** 2023-11-01

**Authors:** Dezy Singh, Ramesh C Tiwari, Arvind Kumar, Ashish R Bhute, Ravi P Meshram, Bhawana Mittal

**Affiliations:** 1 Department of Toxicology and Medical Jurisprudence (Agad Tantra Evam Vidhi Vaidyak), Uttarakhand Ayurved University (UAU) Rishikul Campus, Haridwar, IND; 2 Department of Pathology and Laboratory Medicine, All India Institute of Medical Sciences, Rishikesh, IND; 3 Department of Forensic Medicine and Toxicology (FMT), All India Institute of Medical Sciences, Rishikesh, IND

**Keywords:** epidemiology and public health, from india, gross appearance, histopathology examination, liver, manner of death, cause of death, forensic autopsy

## Abstract

Background: The pathological examination of a medicolegal autopsy is a great learning opportunity for a pathologist as well as for a forensic expert, where the cause of death remains unknown. Liver disease epidemiology differs from one geographic area to another.

Material and methods: This was a prospective observational study with 100 medicolegal autopsy cases over a one-year period conducted in the Department of Forensic Medicine and Toxicology (FMT) and Pathology. Representative tissue from the liver was collected in 10% neutral buffered formalin and sent for histopathological examination.

Results: The mean age of the cases was 41.98 ± 15.39 years, and ages ranged from 20 to 90 years with male preponderance. The most common histopathology and gross findings noted were mild to moderate chronic hepatitis (CH) (54%) and fatty change (36%), respectively. There was a significant association (p ≤ 0.05) between histopathology and gross findings, cause, and manner of death.

Conclusion: Gross and histopathological examination of the liver in a medicolegal autopsy has a significant role in ascertaining the cause and manner of death.

## Introduction

A pathological examination is not confined to determining the cause of death; it is also useful in teaching the pathogenesis and demography of disease [1]. It helps in the quality assessment of clinical disease retrospectively and the formation of new preventive measures [2-6]. Clinical and medicolegal autopsies are incomplete without pathological examination. When supplemented with clinical history, gross and histopathological findings may lead to the final cause of death [1]. A wide range of metabolic, chemical, microbiological, and circulatory insults can harm the liver. In some cases, the hepatic involvement is secondary to cardiac decompensation, drunkenness, or "extrahepatic infections," whereas in other cases, the involvement is primary. The liver is referred to as "the custodian of the interior milieu" with good reason [7]. The results of an autopsy can be used to track the cause of death and develop medical strategies that will help one swiftly and effectively revise and reword one's material [2]. When correlated with the cause and mode of death, the spectrum of gross and histopathological findings may lead to the significant association of variables, creating the scope for framing a prophylactic plan for prevention, ascertaining the demography, or revealing any incidental findings [3]. The goal of this research is to uncover all of the potential benefits of pathological examination, including gross and microscopic features, in ascertaining the cause of death in the present scenario.

## Materials and methods

Study design

An observational study was conducted in the Department of Forensic Medicine and Toxicology (FMT) at All India Institute of Medical Sciences (AIIMS) Rishikesh. The Department of FMT received 100 medicolegal cases for autopsy. From March 2022 to March 2023, histopathology samples were transferred to the Department of Pathology at AIIMS Rishikesh. The autopsy and clinical findings were noted in the Department of FMT of AIIMS Rishikesh.

Representative tissue from macroscopically variable areas of a liver was collected in a 10% neutral buffered formalin in the autopsy room. For histopathological analysis, specimens from all of the medicolegal cases were fixed in a 10% neutral buffered formalin solution. Hematoxylin and eosin (H&E) stains were used to stain each section. The weights of all visceral organs were recorded during the autopsy, and then, sections were examined histologically by the Department of Pathology. Special stains, including Ziehl-Neelsen for tubercular bacilli and Fite-Faraco for lepra bacilli, and silver stains such as Grocott methenamine silver along with periodic acid-Schiff for fungal profiles were done whenever required. Gross and histopathological features were studied, and incidental and interesting findings were noted in a brief discussion.

All confidentiality was maintained, and only non-identifiable data of the patient was collected and tabulated for analysis. Additional consent was obtained for taking histological samples from the deceased's relatives.

Sample size

The sample size was calculated using sample-size.net software.

Scientific method

We expected the most common cause of death to be 35% or less (the number of causes of death was 10) to ensure a confidence interval width of ±10% for the proportion. The sample size was calculated to be 87 by normal approximation.

Exact proportion

The sign test (binomial test) was used: 100 cases with α err prob = 0.05, power (1-β err prob) = 0.8, output: lower critical N = 35.0000000, upper critical N = 55.0000000, and actual power = 0.8122881. Actual α = 0.0445975 was included in the study, qualifying for inclusion criteria.

Inclusion and exclusion criteria

The sample included all medicolegal autopsy cases except previously autopsied cases, police encounter deaths, decomposed bodies, and unidentified bodies.

A prospective observational study was conducted in the Department of FMT at AIIMS Rishikesh. One hundred medicolegal cases presented in the Department of FMT for autopsy were taken. After gross inspection and weight measurement, representative histopathological samples were taken from the livers, kept in 10% neutral buffered formalin, and sent to the Department of Pathology at AIIMS Rishikesh between March 2022 and March 2023. Sections were processed, and paraffin tissue embedding was done to prepare the tissue blocks. Sections (5 mm) were cut in a microtome, and all prepared slides were stained with H&E, mounted, and examined microscopically. This study was approved by the Institutional Ethics Committee of All India Institute of Medical Sciences (AIIMS) (vide letter number: AIIMS/IEC/22/16).

## Results

**Figure 1:**
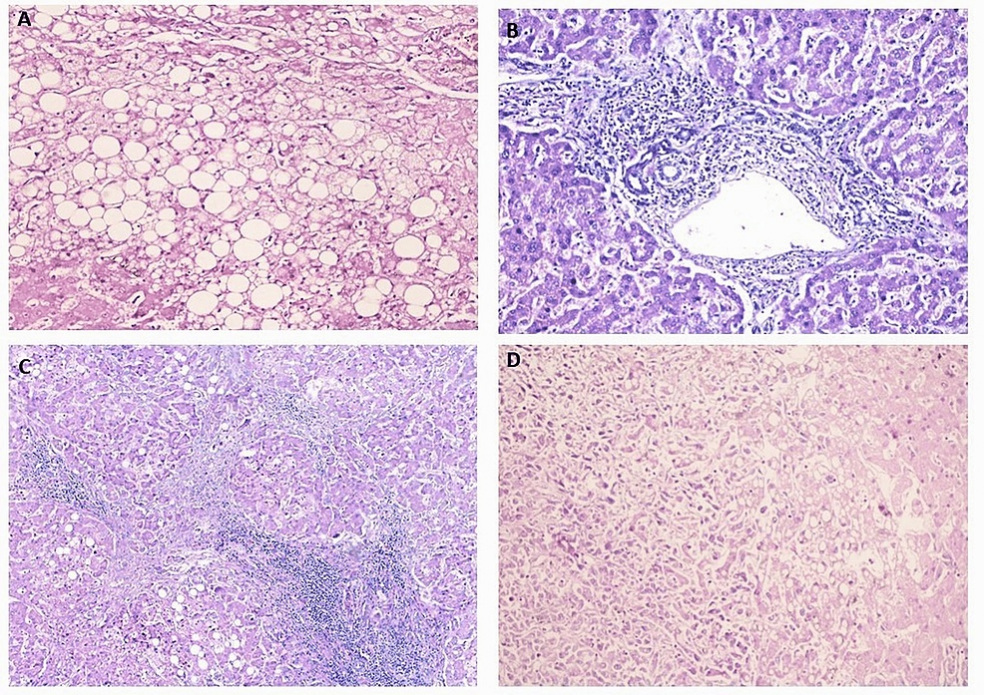
(A) Mixed steatohepatitis with a predominance of macrovesicular change (fatty change) (H&E, 200×), (B) marked sinusoidal dilatation and congestion with moderate interface hepatitis (nutmeg liver) (H&E, 200×), (C) variable hepatic nodule separated by thick fibrous septae (cirrhotic nodules) (H&E, 100×), and (D) infiltrative tumor having oval to spindled atypical cells with vesicular nuclei and a moderate amount of cytoplasm (H&E, 100×) H&E: hematoxylin and eosin

A total of 100 cases were taken in this study with a mean ± standard deviation (SD) age of 41.98 ± 15.39, median (interquartile range (IQR)) of 39.50 (30.00-50.00), and range of 20-90 years, with male preponderance (5.25:1). The most common histopathology (Batts-Ludwig grading and grading for chronic hepatitis (CH)) was mild to moderate chronic hepatitis (54%), including 20 (20%) cases of mild chronic hepatitis (mild CH), 19 (19%) cases of moderate chronic hepatitis (MCH) with steatosis, and 15 (15%) cases of mild CH with steatosis (Figure 1A-1D).

**Figure 2:**
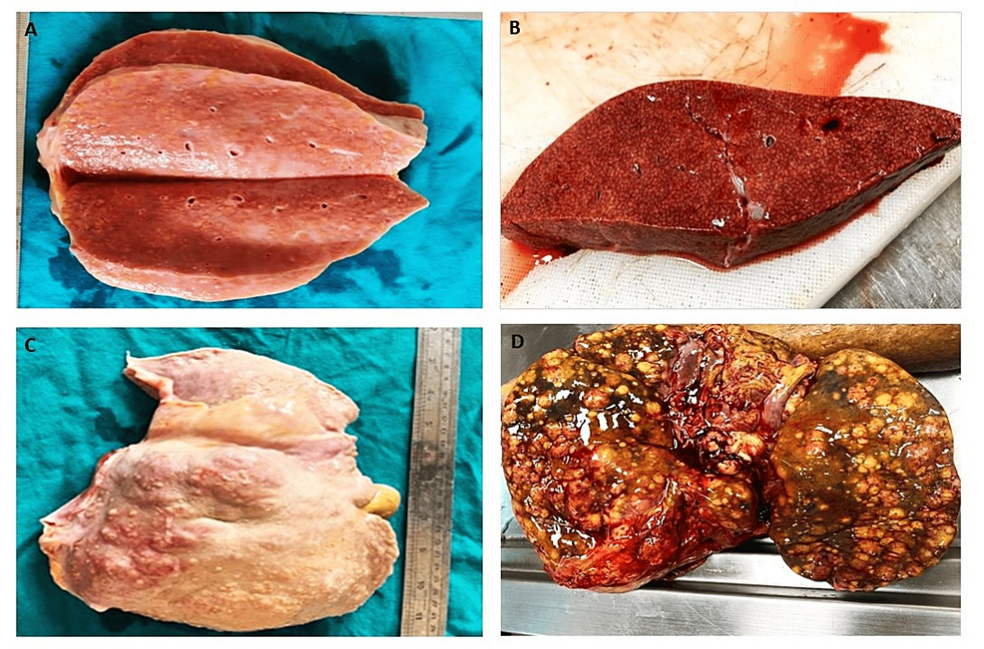
(A) Cut surface shows a yellow greasy surface (fatty change), (B) cut surface shows appearance due to sinusoidal dilatation and congestion (nutmeg liver), (C) outer surface shows variably sized small nodules (cirrhotic nodules), and (D) outer surface of both lobes show numerous variably sized nodules (microscopy revealed malignant tumor)

The most common gross finding was a fatty change (36, 36%), followed by congestion (20, 20%). Eleven (11%) of the cases showed MCH with granuloma. Eleven (11%) of the cases resulted in MCH. Severe CH with definitive cirrhotic nodules was noted in seven (7%) cases (Figure 2).

Five (5%) cases had MCH with cholestasis. Five (5%) cases had mild CH with chronic venous congestion (CVC). Four of the deceased (4%) revealed MCH with necrosis. Three (3%) cases had mild CH with tumors. The mean liver weight was 1431.29 ± 365.48 g. Six (6%) cases had a liver weight of <968 g. Sixty-six (66%) cases weighed 968-1600 g. Twenty-eight (28%) cases weighed >1600 g. Thirty-six (36%) cases had gross findings of fatty change. Twenty (20%) cases had gross findings of congestion. Fourteen (14%) of the autopsy cases showed unremarkable gross findings. Thirteen (13%) of the cases had lacerations. A greenish-yellow cut surface was noted in seven (7%) cases. Four (4%) cases revealed multiple nodules, and three (3%) cases showed multiple space-occupying lesions (SOLs) on gross findings. Three (3%) of the deceased showed a gross finding of nutmeg liver.

A chi-squared test was used to explore the association between liver histopathology and age. There was no significant difference between the various groups in terms of distribution of age (χ2 = 75.170, p = 0.140).

The strength of association between the two variables (Cramer's V) was 0.33 (moderate association). In the group of liver histopathology with MCH and steatosis, 52.6% of the cases were 30-39 years old. In the group with mild CH, 35% of the cases were 40-49 years old (Table 1).

**Table 1:** Association between liver histopathology and age MCH: moderate chronic hepatitis, CH: chronic hepatitis, CVC: chronic venous congestion

Age	Liver histopathology												Chi-squared test	
	Mild CH	MCH + steatosis	Mild CH + steatosis	MCH + granuloma	MCH	Severe CH + definitive cirrhotic nodule	MCH + cholestasis	Mild CH + CVC	MCH + necrosis	Mild CH + tumor	Total	χ2		p-value
20-29 years	3 (15%)	1 (5.3%)	6 (40%)	5 (45.5%)	3 (27.3%)	1 (14.3%)	1 (20%)	1 (20%)	2 (50%)	0 (0%)	23 (23%)	75.170		0.140
30-39 years	4 (20%)	10 (52.6%)	2 (13.3%)	1 (9.1%)	6 (54.5%)	0 (0%)	1 (20%)	1 (20%)	2 (50%)	0 (0%)	27 (27%)			
40-49 years	7 (35%)	3 (15.8%)	4 (26.7%)	2 (18.2%)	1 (9.1%)	3 (42.9%)	1 (20%)	1 (20%)	0 (0%)	0 (0%)	22 (22%)			
50-59 years	3 (15%)	3 (15.8%)	3 (20%)	2 (18.2%)	0 (0%)	2 (28.6%)	1 (20%)	0 (0%)	0 (0%)	0 (0%)	14 (14%)			
60-69 years	1 (5%)	1 (5.3%)	0 (0%)	0 (0%)	1 (9.1%)	1 (14.3%)	0 (0%)	1 (20%)	0 (0%)	2 (66.7%)	7 (7%)			
70-79 years	1 (5%)	0 (0%)	0 (0%)	1 (9.1%)	0 (0%)	0 (0%)	1 (20%)	1 (20%)	0 (0%)	1 (33.3%)	5 (5%)			
80-89 years	0 (0%)	1 (5.3%)	0 (0%)	0 (0%)	0 (0%)	0 (0%)	0 (0%)	0 (0%)	0 (0%)	0 (0%)	1 (1%)			
≥90 years	1 (5%)	0 (0%)	0 (0%)	0 (0%)	0 (0%)	0 (0%)	0 (0%)	0 (0%)	0 (0%)	0 (0%)	1 (1%)			
Total	20 (100%)	19 (100%)	15 (100%)	11 (100%)	11 (100%)	7 (100%)	5 (100%)	5 (100%)	4 (100%)	3 (100%)	100 (100%)			

There was a significant difference between the various groups in terms of the distribution of cause of death (χ2 = 149.777, p = <0.001). The strength of association between the two variables (Cramer's V) was 0.41 (moderate association). In the group histopathology MCH with cholestasis, the cause of death for 80% of the cases was brain injury. In the group histopathology MCH with necrosis, the cause of death for 75% of the cases was poisoning. In the group histopathology with mild CH with CVC, the cause of death in 60% of cases was antemortem hanging (Table 2).

**Table 2:** Association between liver histopathology and cause of death MCH: moderate chronic hepatitis, CH: chronic hepatitis, CVC: chronic venous congestion

Cause of death	Liver histopathology											Chi-squared test	
	Mild CH	MCH + steatosis	Mild CH + steatosis	MCH + granuloma	MCH	Severe CH + definitive cirrhotic nodule	MCH + cholestasis	Mild CH + CVC	MCH + necrosis	Mild CH+ tumor	Total	χ2	p-value
Brain injury	6 (30%)	11 (57.9%)	6 (40%)	3 (27.3%)	4 (36.4%)	1 (14.3%)	4 (80%)	0 (0%)	0 (0%)	0 (0%)	35 (35%)	149.777	<0.001
Septicemia	1 (5%)	1 (5.3%)	4 (26.7%)	1 (9.1%)	4 (36.4%)	3 (42.9%)	0 (0%)	1 (20%)	0 (0%)	1 (33.3%)	16 (16%)		
Hemorrhagic shock	2 (10%)	0 (0%)	1 (6.7%)	5 (45.5%)	0 (0%)	0 (0%)	0 (0%)	0 (0%)	1 (25%)	0 (0%)	9 (9%)		
Unclear	5 (25%)	1 (5.3%)	1 (6.7%)	0 (0%)	0 (0%)	1 (14.3%)	0 (0%)	1 (20%)	0 (0%)	0 (0%)	9 (9%)		
Miscellaneous	3 (15%)	1 (5.3%)	0 (0%)	2 (18.2%)	0 (0%)	1 (14.3%)	0 (0%)	0 (0%)	0 (0%)	1 (33.3%)	8 (8%)		
Antemortem hanging	1 (5%)	2 (10.5%)	0 (0%)	0 (0%)	0 (0%)	0 (0%)	1 (20%)	3 (60%)	0 (0%)	0 (0%)	7 (7%)		
Poisoning	0 (0%)	1 (5.3%)	2 (13.3%)	0 (0%)	1 (9.1%)	0 (0%)	0 (0%)	0 (0%)	3 (75%)	0 (0%)	7 (7%)		
Coronary artery disease	2 (10%)	1 (5.3%)	0 (0%)	0 (0%)	0 (0%)	1 (14.3%)	0 (0%)	0 (0%)	0 (0%)	0 (0%)	4 (4%)		
Pneumonia	0 (0%)	1 (5.3%)	1 (6.7%)	0 (0%)	1 (9.1%)	0 (0%)	0 (0%)	0 (0%)	0 (0%)	0 (0%)	3 (3%)		
Pulmonary edema	0 (0%)	0 (0%)	0 (0%)	0 (0%)	1 (9.1%)	0 (0%)	0 (0%)	0 (0%)	0 (0%)	1 (33.3%)	2 (2%)		
Total	20 (100%)	19 (100%)	15 (100%)	11 (100%)	11 (100%)	7 (100%)	5 (100%)	5 (100%)	4 (100%)	3 (100%)	100 (100%)		

**Figure 3:**
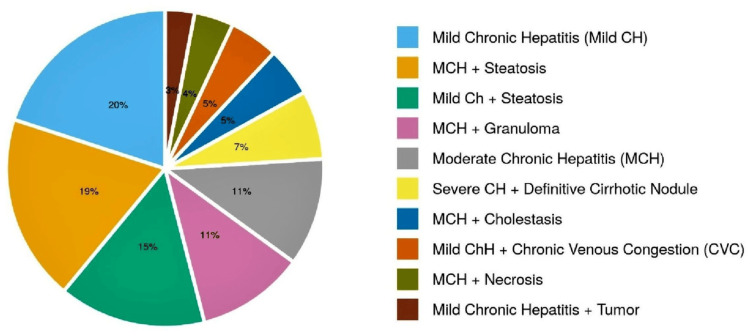
Distribution of histopathological findings MCH: moderate chronic hepatitis, CH: chronic hepatitis, CVC: chronic venous congestion

Figure 3 shows the distribution of histopathological findings.

There was a significant difference between the various groups in terms of the distribution of manner of death (χ2 = 44.048, p = 0.020). The strength of association between the two variables (Cramer's V) was 0.38 (moderate association). In the group histopathology with mild CH, the manner of death was accidental in 80% of the cases. In the group histopathology MCH with necrosis, the manner of death was suicide in 75% of cases. In the group histopathology MCH with cholestasis, the manner of death was accidental in 80% of cases (Table 3).

**Table 3:** Association between liver histopathology and manner of death MCH: moderate chronic hepatitis, CH: chronic hepatitis, CVC: chronic venous congestion

Manner of death	Liver histopathology											Chi-squared test	
	Mild CH	MCH + steatosis	Mild CH + steatosis	MCH + granuloma	MCH	Severe CH + definitive cirrhotic nodule	MCH + cholestasis	Mild CH + CVC	MCH + necrosis	Mild CH + tumor	Total	χ2	p-value
Accidental	16 (80%)	14 (73.7%)	8 (53.3%)	10 (90.9%)	7 (63.6%)	4 (57.1%)	4 (80%)	2 (40%)	1 (25%)	1 (33.3%)	67 (67%)	44.048	0.020
Natural	3 (15%)	2 (10.5%)	5 (33.3%)	0 (0%)	3 (27.3%)	3 (42.9%)	0 (0%)	0 (0%)	0 (0%)	2 (66.7%)	18 (18%)		
Suicidal	1 (5%)	2 (10.5%)	2 (13.3%)	1 (9.1%)	1 (9.1%)	0 (0%)	1 (20%)	3 (60%)	3 (75%)	0 (0%)	14 (14%)		
Homicidal	0 (0%)	1 (5.3%)	0 (0%)	0 (0%)	0 (0%)	0 (0%)	0 (0%)	0 (0%)	0 (0%)	0 (0%)	1 (1%)		
Total	20 (100%)	19 (100%)	15 (100%)	11 (100%)	11 (100%)	7 (100%)	5 (100%)	5 (100%)	4 (100%)	3 (100%)	100 (100%)		

**Figure 4:**
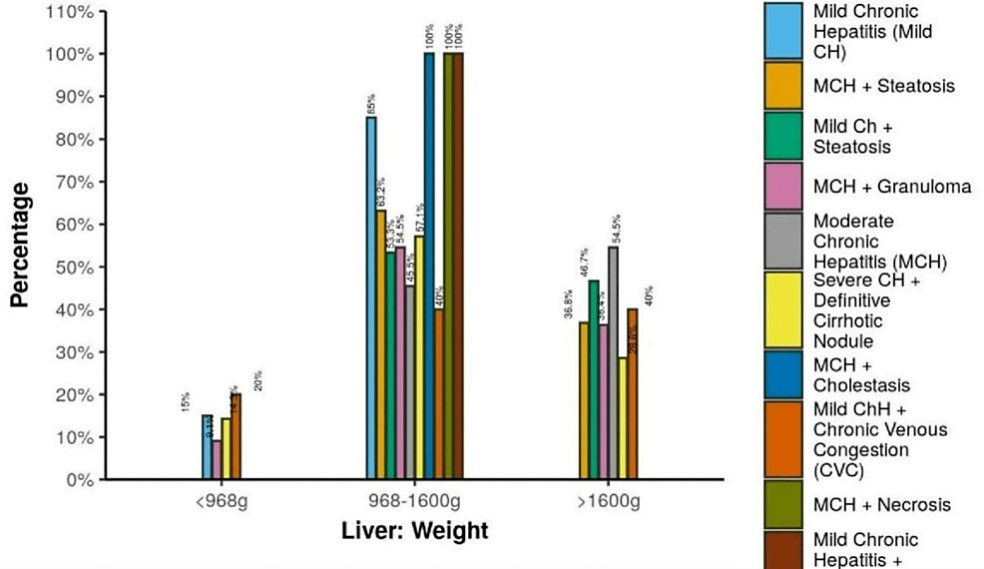
Association between liver histopathology and liver weight (g) MCH: moderate chronic hepatitis, CH: chronic hepatitis, CVC: chronic venous congestion

Figure 4 shows the association between liver histopathology and liver weight (g).

There was a significant difference between the 10 groups in terms of liver weight (g) (χ2 = 22.515, p = 0.007), with the median weight (g) being highest in the liver histopathology MCH group. The strength of association (Kendall's tau) was 0.16 (small effect size) (Table 4).

**Table 4:** Association between liver histopathology and liver weight (g) MCH: moderate chronic hepatitis, CH: chronic hepatitis, CVC: chronic venous congestion, SD: standard deviation, IQR: interquartile range

Liver weight (g)	Liver histopathology										Kruskal-Wallis test	
	Mild CH	MCH + steatosis	Mild CH + steatosis	MCH + granuloma	MCH	Severe CH + definitive cirrhotic nodule	MCH + cholestasis	Mild CH + CVC	MCH + necrosis	Mild CH + tumor	χ2	p-value
Mean (SD)	1161.50 (203.99)	1467.32 (352.48)	1644.00 (352.72)	1454.55 (487.86)	1603.64 (327.42)	1400.00 (297.99)	1258.00 (209.09)	1510.00 (594.64)	1515.00 (58.02)	1340.00 (262.87)	22.515	0.007
Median (IQR)	1120 (1020-1350)	1430 (1165-1760)	1540 (1380-1870)	1260 (1080-1845)	1680 (1510-1880)	1390 (1250-1575)	1230 (1080-1420)	1440 (1140-1980)	1535 (1505-1545)	1450 (1245-1490)		
Minimum-maximum	850-1540	980-2050	1100-2340	890-2390	980-1930	950-1810	1040-1520	770-2220	1430-1560	1040-1530		

**Figure 5:**
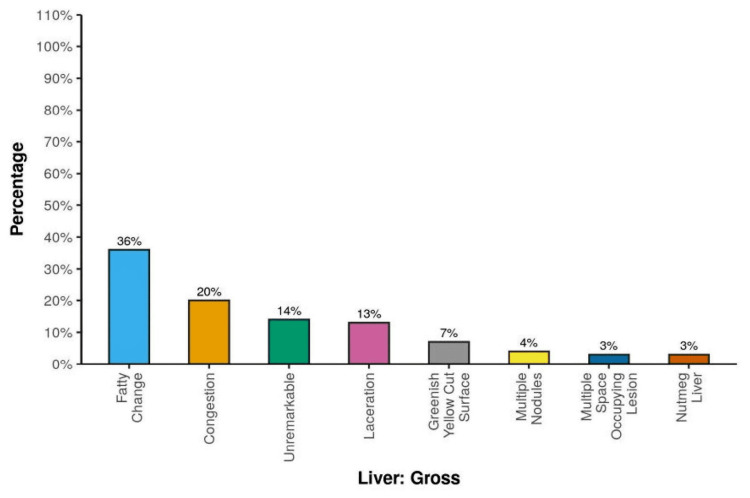
Distribution of gross findings

In the group histopathology MCH with cholestasis, 100% of the cases had liver weights between 968 and 1600 g. In the group of histopathology MCH with necrosis, 100% of the cases had weights between 968 and 1600 g. In the group histopathology mild chronic hepatitis with tumors, 100% of the cases had weights between 968 and 1600 g (Figure 5).

There was a significant difference between the various groups in terms of the distribution of liver gross (χ2 = 311.805, p ≤ 0.001). In the group with liver histopathology mild chronic hepatitis with tumors, 100% of the cases had multiple SOLs on gross findings. In the group histopathology MCH with granuloma, 90.9% of the cases had gross findings of laceration (Table 5).

**Table 5:** Association between liver histopathology and liver gross MCH: moderate chronic hepatitis, CH: chronic hepatitis, CVC: chronic venous congestion

Liver gross	Liver histopathology											Chi-squared test	
	Mild CH	MCH + steatosis	Mild CH + steatosis	MCH + granuloma	MCH	Severe CH + definitive cirrhotic nodule	MCH + cholestasis	Mild CH + CVC	MCH + necrosis	Mild CH + tumor	Total	χ2	p-value
Fatty change	2 (10%)	11 (57.9%)	12 (80%)	1 (9.1%)	5 (45.5%)	2 (28.6%)	0 (0%)	0 (0%)	3 (75%)	0 (0%)	36 (36%)	311.805	<0.001
Congestion	10 (50%)	2 (10.5%)	1 (6.7%)	0 (0%)	2 (18.2%)	0 (0%)	0 (0%)	4 (80%)	1 (25%)	0 (0%)	20 (20%)		
Unremarkable	8 (40%)	3 (15.8%)	0 (0%)	0 (0%)	2 (18.2%)	0 (0%)	1 (20%)	0 (0%)	0 (0%)	0 (0%)	14 (14%)		
Laceration	0 (0%)	1 (5.3%)	1 (6.7%)	10 (90.9%)	1 (9.1%)	0 (0%)	0 (0%)	0 (0%)	0 (0%)	0 (0%)	13 (13%)		
Greenish-yellow cut surface	0 (0%)	2 (10.5%)	1 (6.7%)	0 (0%)	0 (0%)	1 (14.3%)	3 (60%)	0 (0%)	0 (0%)	0 (0%)	7 (7%)		
Multiple nodules	0 (0%)	0 (0%)	0 (0%)	0 (0%)	0 (0%)	4 (57.1%)	0 (0%)	0 (0%)	0 (0%)	0 (0%)	4 (4%)		
Multiple space-occupying lesion	0 (0%)	0 (0%)	0 (0%)	0 (0%)	0 (0%)	0 (0%)	0 (0%)	0 (0%)	0 (0%)	3 (100%)	3 (3%)		
Nutmeg liver	0 (0%)	0 (0%)	0 (0%)	0 (0%)	1 (9.1%)	0 (0%)	1 (20%)	1 (20%)	0 (0%)	0 (0%)	3 (3%)		
Total	20 (100%)	19 (100%)	15 (100%)	11 (100%)	11 (100%)	7 (100%)	5 (100%)	5 (100%)	4 (100%)	3 (100%)	100 (100%)		

## Discussion

Monitoring the cause of death and developing a medical strategy are both aided by autopsy studies [7]. The causes of many liver ailments are well established. Three pathologically distinct liver disorders are typically brought on by excessive alcohol consumption: fatty liver, hepatitis, and alcoholic cirrhosis. Any of the three may occur simultaneously in the same patient or even all three [8].

A vast range of liver diseases have been seen in liver autopsies, and they play a large part in mortality all over the world [9].

In the present study, 100 cases of medicolegal autopsies were done; the most common gross finding of the liver was fatty change (36%), followed by congestion (20%), with a mean age of 39.61 ± 13.20 and 40.35 ± 13.50, respectively (Figure 2A, 2B). Similar gross results were obtained from different geographic areas by Bal et al. [7], Kulkarni et al. [10], Thamil Selvi et al. [11] from Tamil Nadu, and Umesh et al. from Karnataka [12].

Clinicians must have a high index of suspicion because serious liver disorders might be asymptomatic and may not be detected until death. Therefore, it is crucial that clinicians precisely assess the liver's functioning condition in order to promptly identify the disease for effective treatment [13].

Association between histopathology and weight and gross finding

The weight of an organ can reveal its underlying diseased state. Some clinical disorders lead to an increase in organ weight, while other conditions result in organ shrinkage and weight loss. Organ weight can provide a crucial clue in distinguishing a diseased organ from a healthy one.

The mean weight of a liver in our study was 1431.29 ± 365.48 g. Six (6%) cases had a liver weight of <968 g. Sixty-six (66%) cases had a liver weight of 968-1600 g. Twenty-eight (28%) cases had a liver weight of >1600 g. Similar gross results were obtained by Prakash et al. [14] from Uttarakhand, Singh et al. [15] from Chandigarh, Kohli et al. [16] from Delhi, and John et al. [17] from Kerala.

From this study, we can conclude that the liver weights were higher in males than in females. There are no significant differences in the organ weights obtained in our studies compared to other studies conducted in India (Table 4) [14-16].

There was gross and histopathological concordance regarding fatty change, since there was 36% fatty change on gross finding (cut surface) of the liver, and microscopy shows 34% fatty change on representative sections (Table 5).

Association between manner of death and histopathology

Suicide as the manner of death comprised 14% of the cases. They are significantly associated (p < 0.020) with liver histopathology revealing mild CH to MCH with CVC and necrosis in 42.8% of cases (Table 3). This study is comparable to the studies of Umesh et al. [12].

Rare gross finding

We had a rare single case of left lobe absence found incidentally in a 55-year-old male. Agenesis of the left hepatic lobe is a rare anomaly. It is usually noted incidentally at autopsy or surgery. Clinically, most patients without right or left hepatic lobe are generally asymptomatic. Few cases have been described in the literature regarding the frequency of agenesis of the left hepatic lobe [18-22]. Sato et al. [23] reported a congenital agenesis of the right lobe of the liver detected in a patient with gastric cancer. Ceravolo et al. [24] reported a rare case of left liver lobe absence in an 80-year-old male patient discovered during an MRI scan.

Association between multiple nodule findings and age 

Multiple nodules on gross findings lead to the differential diagnosis of cirrhosis and malignancy (Figure 2C, 2D). Histopathology was conclusive in that scenario. Hepatic cirrhosis is the end stage of several hepatopathies [25]. The new global burden of disease estimates for liver cirrhosis, published in BMC Medicine, suggest that cirrhosis caused over a million deaths in 2010, with a further million due to liver cancer and acute hepatitis [26]. Only four (4%) cases exhibited the gross finding of multiple nodules in both lobes with a mean age of 41.50 ± 13.77, with more in the age group of 40-49 in year 2 (50%) (Table 1). Machnik et al. [27] and Watanabe et al. [28] obtained similar gross results from different geographic areas. In the present study, histopathological findings in severe CH with definitive cirrhotic nodule showed seven (7%) cases with a mean age of 46.57 ± 13.15 and a male-to-female ratio of 6:1.

The combination of a high incidence of cirrhosis and the increasing average age of patients will probably result in an increased occurrence of hepatocellular carcinoma [29].

Because major liver diseases may be asymptomatic and may not be diagnosed until death, there is a need for a high index of suspicion by clinicians [30].

Liver laceration finding

A liver injury resulting from blunt trauma is the second leading cause of death, and the liver is the most common organ to get injured from penetrating injuries [31]. Road traffic accidents (RTAs) are among the top five causes of morbidity and mortality in Southeast Asian countries [32].

RTA rates in India are some of the highest in the world and are reported to be 20 times more than those reported in developed countries [33].

Livers are the most often injured organs in RTAs according to studies by Chaudhary et al. [34] and Subedi et al. [35], contrary to the findings of Shetty et al. [36] and Bakkannavar et al. [37], in which they concluded that kidneys were the most commonly affected organ in trauma cases.

According to Khajuria et al. [38] and Farooqui et al. [39], the most commonly injured organ is the brain. According to Ravindra et al. [40] and Asuquo et al. [41], the spleen is the most commonly injured organ. Among the 100 autopsies performed in the present study, 67 (67%) were RTAs and other mechanical traumatic cases. Some forms of liver injuries were noted in 13 cases on gross inspection with a male-to-female ratio of 12:1. Their mean age was 39.08 ± 16.18. The most common age range of the victims was 20-39 years, followed by the age group 30-39 years. This is in accordance with the study done by Reddy et al. [42] from Maharashtra, Wardha, with an age group of 21-40 years and a male-to-female ratio of 11.5:1, with 32% liver damage in the form of lacerations in a duration of two years. According to Reddy et al. [42], the lungs are the most commonly injured organ in RTAs.

Studies from Rwanda, Iran, and Nepal have validated this finding of male predominance and younger age among RTA victims [43-45]. The age group most affected was 16-30 years old (47.4% cases). Other studies from various parts of India have also shown that a majority of the victims belonged to the 20-29 or 20-30 age group [46]. Blunt abdominal trauma is one of the leading causes of mortality among trauma victims. It is the main cause of death in people under 35 years of age worldwide [47]. There is a need to take more consolidated safety measures and implement strict traffic rules and risk stratification in the susceptible population to educate the people [48].

Association between cause of death and liver histopathology

Regarding the cause of death due to brain injury, 31.4% had histopathology of MCH with steatosis, followed by 17.1% histopathology of mild CH, which was statistically correlated (p ≤ 0.001). There was a significant difference between the various groups in terms of the distribution of histopathology (χ2 = 149.777, p ≤ 0.001) (Table 2).

Because major liver diseases may be asymptomatic and may not be diagnosed until death, there is a need for a high index of suspicion by clinicians. Therefore, it is important for clinicians to properly assess the functional state of the liver to prevent, detect, and promptly treat these disorders [49].

Association between cause of death and gross finding

In our gross study, brain injury was the most common cause of death, followed by septicemia. The total cases of brain injury included 35 cases; 34.3% of the gross findings of cases showed brain injury as the cause of death. In 37.5% of the cases in the group, septicemia as the cause of death revealed fatty changes on gross findings (statistically correlated) (p = 0.014).

There was a significant difference between the various groups in terms of the distribution of liver gross (χ2 = 90.175, p = 0.014) (Table 5).

Statistical analysis revealed a significant association between brain injury and fatty liver change. As we know, these are independent variables; their statistically significant association signifies that these findings might be incidental and indirectly throw light on the deranged lifestyle of the individuals, which was responsible for the liver diseases in variable grades.

Cause of death: Septicemia

Septicemia is the second most common cause of death, statistically correlating (p < 0.001) with histopathology exhibiting mild CH to MCH with steatosis in 50% of cases. Garofalo et al. [50] described nonspecific histopathological findings such as steatosis, cholangiolitis, intrahepatic cholestasis, periportal inflammation, and apoptosis. Few studies concluded that steatosis in patients with sepsis [51-53] can be due to the effect of bacterial toxins that may cause macrovesicular [54] or microvesicular steatosis [55].

Specific histological findings fail to correlate to the various clinical manifestations of sepsis-induced organ dysfunction. Further studies are needed to establish this relationship. However, the procurement of tissue samples, necessarily from postmortem studies, imposes an insurmountable difficulty for the design of these studies [56].

Cause of death: Poison

Cause of death due to poison constituted 7% of cases significantly associated with MCH, with necrosis on histopathology in 43% of cases. This finding is consistent with studies by Sinha et al. [57], Arora et al. [58], and Ghazi [59] depicting congestion, fatty infiltration, and centrizonal necrosis in liver histopathology. Aluminum phosphide, also known as "wheat pill," is a poison that can be fatal and has quick-moving symptoms that lead to death. Therefore, early evacuation to a tertiary healthcare facility with adequate supporting care is a successful strategy.

Space-occupying lesions

The liver is the common site for space-occupying lesions (SOLs). In the present study, three cases of SOLs were noted in three males with a mean age of 67.33 ± 4.04.

There were two (66.7%) cases at age 60-69, followed by one (33.3%) case at age 70-79. These lesions were incidental findings, and they were confirmed only on histopathology (statistically correlated) (p = 0.0251). In a study by Sonarkar et al. [60], the common age group affected was 50-60 years. Sheefa et al. [61] and Edris et al. [62] showed a mean age of 55.5, 57.7, and 59.4 years. In the present study, the most commonly affected age group was 60-69 years. Space-occupying lesions of the liver were commonly observed in the fifth decade of life. Males were affected more than females. Metastatic lesions of the liver were the most common cause of liver SOLs (34.9%), followed by liver abscesses [63].

The gross diagnosis made at the time of autopsy, particularly in the heart, lungs, liver, and kidneys, is greatly influenced by histological investigation. Adequate sampling and histological analysis are important for autopsy quality. Even in the era of high-tech medicine, pathological examination in autopsy remains an important tool for the quality assessment of clinical diagnosis. Various findings unrelated to the cause of death may be noted during the histopathological examination of various organs. Most missed gross diagnoses are due to the subtleness and variety of disease processes, as several significant illnesses might exist without typical clinical signs.

Highlights of the study

There was a significant statistical correlation between histopathology and poisoning as the cause of death and antemortem hanging cases. The manner of death, especially natural and suicidal, had a significant association with histopathology. Most cases of cirrhosis and incidental malignancy were found in the 40-59 years and 60-79 years age group, respectively, depicting the demography of the disease. Road traffic accidents were the most common cause of demise in the study.

Future scope of the study

Multi-institutional studies with multiple organ systems and large sample sizes can consolidate the interpretation of pathological findings in arriving at the cause of death. It can also delineate the demography of the disease and hence will help formulate preventive measures in that region. Tissues from the autopsy can serve the purpose of framing the structure of biobanking in research. Studies can also enrich the forensic pathological acumen of postgraduate students of forensic medicine and pathology.

Limitations of the study

The study findings are limited by its small sample size and including only one organ. It could have been better to take a more representative section of the organ, delineating the whole gross picture. History of comorbidities and relevant past history were lacking in the study. They could be used in the consolidated association between pathological findings and cause of death.

## Conclusions

Liver histopathology is significantly associated with age, cause of death, manner of death, liver weight, and gross findings. Gross and histopathological findings played a vital role in establishing the cause and mode of death. However, histopathology emerged as an undisputed tool to lead to the final cause of death. Disease demography and incidental findings also depend on the histopathology of organ autopsy.
